# Quasi-Spatially Periodic Signals for Optical Fibre Communications

**DOI:** 10.1038/s41598-018-33589-3

**Published:** 2018-10-22

**Authors:** Qun Zhang, Terence H. Chan, Alex Grant

**Affiliations:** 1 University of South Australia, Institute for Telecommunications Research, Mawson Lakes, SA 5095 Australia; 2 University of Toronto, The Edward S. Rogers Sr. Department of Electrical and Computer Engineering, Toronto, ON M5S 3G4 Canada; 3 Myriota, Adelaide, SA 5000 Australia

## Abstract

We study quasi-spatially periodic signals (QSPSs) as a class of input signals of interest, which maintain their shapes quasi-periodically (with a phase change and a time shift) during propagation in an optical fibre. Instead of the computationally expensive nonlinear Fourier transform (NFT), the property of quasi-periodic shape invariant could be used as an alternative for decoding at the receiver. In this paper, properties of QSPSs and the signal design problem are studied, including the trade-off between various system parameters.

## Introduction

In the ever increasing demand for data traffic, which grows at a pace of 60% per year^1^, fibre optical communication plays a significant role in modern information technological infrastructure. Compared to copper wires, optical fibres are promising media, especially for long distance data transmission, because they provide ultra high speed and low loss transmission. The fibre loss is only 0.2 dB/km, which means that we only loss half of the signal power after a 15 km propagation.

Although the desirable communication capability an optical fibre has, its physical properties are unfortunately complicated. The signal transmission through it is nonlinear, which makes it difficult to analyse. The traditional tools developed for linear time-invariant (LTI) systems, such as signal detection and error control coding, do not work well in optical fibre channels. The reason is that the underlying physics of nonlinear fibre-optic channels are not satisfied by the “flawed” traditional linear approaches. The fibre nonlinearity could be approximated as a small perturbation in a low signal-to-noise ratio (SNR) regime, however, becomes significant when the SNR is high. As a result, fibre nonlinearity is treated as a detrimental effect in existing applications.

To meet the rapidly increasing demand for the data transmission bandwidth, many multiplexing techniques such as optical time division multiplexing (OTDM)^2^, wavelength division multiplexing (WDM)^3,4^, and orthogonal frequency division multiplexing (OFDM)^5^ have been proposed. However, interference between different users is inevitable under those multiplexing techniques. Take the WDM for an example, it was shown^4^ that the spectral efficiency (SE) for an optical fibre network increases at the low SNR regime, similar to the trend of the linear capacity bound, however, decreases when the signal power is further increased. The reason for the unnatural SE decrease in the high SNR regime is that the interference between different users increases significantly at the high input power regime, which decreases the capacity. In order to tackle this issue, Yousefi and Kschischang recently proposed the nonlinear frequency division multiplexing (NFDM)^6–8^ via the use of the nonlinear Fourier transform (NFT)^9^ (also known as the direct scattering transform^10^). The fibre nonlinearity and the group velocity dispersion (GVD) effects are fully characterised in integrable nonlinear optical fibre communication systems in the nonlinear spectral domain with the help of the NFT. Under the NFDM, a fibre optical channel is subdivided to multiple multiplicative sub-channels in the nonlinear frequency domain. As a result, interference among users can be minimised. Some progresses of the NFT-based communications can be found in^11–16^.

Signal propagation along an optical fibre is characterised by the normalised nonlinear Schrödinger equation (NLSE)^17^

1 $$j\frac{\partial q(t,z)}{\partial z}=\frac{{\partial }^{2}q(t,z)}{\partial {t}^{2}}+2|q(t,z){|}^{2}q(t,z).$$

Here, $j=\sqrt{-\,1}$, *z* and *t* are spatial and temporal variables respectively, and *q*(*t*, *z*) is the complex envelope of the normalised electrical field after propagating a distance *z* in the optical fibre. We note that fibre loss is assumed to be perfectly compensated by the ideal distributed Raman amplification in (1). However, the scenario of lumped amplification was also studied previously. For example, the NLSE with periodic perturbation was studied^18^, in which the robustness of the solitons suffered from periodic perturbations was characterised by the resonance condition related to the soliton period. The periodic perturbations^18^ could be due to the lumped amplifications applied repeatedly every certain distance along the transmission.

For each fixed $z\in {\mathbb{R}}$, the NFT of *q*(*t*, *z*) consists of continuous and discrete parts:

$$\{{Q}^{(c)}(\lambda ,z):\,\lambda \in {\mathbb{R}})\}\,{\rm{and}}\,\{{Q}^{(d)}({\zeta }_{k},z),{\zeta }_{k}\in {{\mathbb{C}}}^{+}:k=1,2,\ldots ,N\}.$$

We call $\{\lambda :\,\lambda \in {\mathbb{R}}\}$ and *ζ*_*k*_ the continuous spectrum and a discrete eigenvalue, while *Q*^(*c*)^(*λ*, *z*) and *Q*^(*d*)^(*ζ*_*k*_, *z*) their corresponding continuous spectral function and discrete spectral amplitudes. In this paper, we assume that the discrete eigenvalues are simple zeros of the associated scattering function. For detailed treatment of the NFT, please refer to^10,19,20^. Fast algorithms for the NFT and its inverse were studied in^21–23^. Recently, an alternative decoding method^24^ is proposed, which directly uses scattering data instead of solving the eigenvalues.

In the NFDM, encoding is conducted in the nonlinear frequency domain. Each user is allocated a portion of nonlinear frequency spectrum (discrete and/or continuous), over which data is encoded and modulated. To illustrate, assume that there is only one user (and hence it has exclusive use of all the nonlinear spectrum). The encoding function *f* is defined over the set $\{1,\ldots ,{2}^{R}\}$ such that for a given set of eigenvalues $\{{\zeta }_{k}:\,k=1,2,\ldots ,N\}\subsetneq {{\mathbb{C}}}^{+}\triangleq$ $\{c\in {\mathbb{C}}:{\rm{Im}}(c) > 0\}$,

$$f(i)=({x}_{k,i},k=1,2,\ldots ,N),\,i\in \{1,\ldots ,{2}^{R}\},$$

where ${x}_{k,i}\in {\mathbb{C}}$ is a spectral amplitude, and *R* is the number of bits per transmission in each symbol. The physical interpretation for *f* is very simple. If the transmitted symbol is *i*, then the channel input is a function *q*(*t*, 0) with spectrum $\{{\zeta }_{k}:\,k=1,2,\ldots ,N\}\subsetneq {{\mathbb{C}}}^{+}$, and spectral function (amplitudes)

$$\begin{array}{ll}{Q}^{(c)}(\lambda ,0)=0, & \forall \,\lambda \in {\mathbb{R}};\\ {Q}^{(d)}({\zeta }_{k},0)={x}_{k,i}, & {\zeta }_{k}\in {{\mathbb{C}}}^{+},\,k=1,2,\ldots ,N.\end{array}$$

To transmit, the signals designed in the nonlinear frequency domain are transformed into their time-domain forms by the inverse nonlinear Fourier transform (INFT), and be subsequently transmitted along the fibre. As the continuous spectrum is null, obtaining the INFT is reduced to solving a set of algebraic equations. At receiver, the received time domain signals are first converted into their spectral domain representations by the NFT. Users can then decode their messages by extracting the portion of the signals in their allocated spectra.

We notice that the computationally expensive NFT (and also its inverse) are involved in the signal de/modulation. In this paper, we consider the class of quasi-spatially periodic signals (QSPSs, whose definition will be given shortly), which guarantees that the “shape” of the channel output signal remains largely the same as that of the input. In this paper, our aim is to report this interesting property, which suggests a potential alternative decoding method instead of the NFT.

To illustrate the concept, we consider the following example. Let the length of an optical fibre be ${\mathfrak{L}}$. (In some application scenarios, the length of a fibre is fixed and known, e.g. fibres in chips). Suppose the possible channel inputs are *q*_1_(*t*, 0) = *β* sech(*βt*), *q*_2_(*t*, 0) = 2*β* sech(*βt*), and *q*_3_(*t*, 0) = 3*β* sech(*βt*) with discrete spectrum respectively {*jβ*/2}, {*jβ*/2, 3*jβ*/2}, and {*jβ*/2, 3*jβ*/2, 5*jβ*/2} where $\beta =\sqrt{k\pi /4{\mathfrak{L}}}$ and $k\in {{\mathbb{Z}}}^{+}$. As we shall see, the shapes of the three signals remain unchanged at the receiver. Therefore, to decode (i.e., to determine which one of the three signals is actually transmitted), one can use time-domain signal processing technique to achieve this goal.

Fundamental solitons (or abbreviated as solitons)^25^ are examples for the QSPSs. Solitons have been studied extensively in the literature in various aspects such as noise modelling^8,26–30^, capacity analysis^26,31,32^ and Gordon-Haus effect characterising the arrival time jitter of a soliton^33^ etc. However, there is only a limited set of soliton solutions. It will therefore be useful, and is also a goal of this paper, to determine a wider class of QSPSs. As we shall see, all QSPSs are *N*-solitons^15,34^ (but not vice versa). In particular, we identify conditions for when an *N*-soliton is quasi-spatially periodic. Signal design is also studied.

The contributions of this paper are as follows. First of all, the QSPS is defined, and its spectral composition is analysed. Using the theorem we derived, it is easy to identify whether a given spectrum corresponds to a QSPS. Furthermore, we propose a trade-off between various system parameters, including minimum period, signal degree-of-freedom, and signal energy constraint. Compared to our earlier conference version^35^, we broaden the notion of the periodicity by allowing both a time shift and a phase change of the signal in each period of distance, and consider a practical problem of the effect of the time domain pulse truncation for the QSPSs.

In this paper, we consider vanishing inputs, i.e.

2 $$\mathop{\mathrm{lim}}\limits_{|t|\to 0}\,q(t,0)=0,\,q(t,0)\in {L}^{1}({\mathbb{R}}).$$

We summarise notations as follows. Denote the NFT of a signal *q*(*t*, *z*), $z\in {\mathbb{R}}$ by (*Q*^(*c*)^(*λ*, *z*), *Q*^(*d*)^(*ζ*_*k*_, *z*)), where $\lambda \in {\mathbb{R}}$ is its continuous spectrum, and ${\zeta }_{k}\in {{\mathbb{C}}}^{+}$, $k=1,2,\ldots ,N$ are eigenvalues which form its discrete spectrum. *Q*^(*c*)^(*λ*, *z*) and *Q*^(*d*)^(*ζ*_*k*_, *z*) are respectively its continuous and discrete spectral amplitudes.

## Results

This section is organised as follows. We first present the necessary and sufficient condition for a signal to be quasi-spatially periodic in the first subsection. Then we discuss the problem of signal design, including the trade-off between various parameters.

### Quasi-Spatially Periodic Signals

In this subsection, we define quasi-spatially periodic signals (QSPSs), and study their properties. In particular, we obtain the relation between the minimum period of a QSPS and its spectrum.

*Concept of Quasi-Spatially Periodic Signals*.

#### **Definition 1**

(Quasi-Spatially Periodic Signals). *A signal q*(*t*, *z*) *propagated in an optical fibre is called a QSPS with period p* > 0 *if for any* ${z}_{0}\in {\mathbb{R}}$, *there exist real numbers t*_0_ *and ϕ such that*

3 $$q(t,{z}_{0}+p)=q(t-{t}_{0}({z}_{0}),{z}_{0}){e}^{j\varphi ({z}_{0})},\,\forall \,t\in {\mathbb{R}}.$$

Note that, *ϕ* in (3) is not unique. If *ϕ* satisfies (3), then *ϕ* + 2*kπ* also satisfies (3) for any integers *k*. Therefore, we may assume without loss of generality that 0 ≤ *ϕ* < 2*π*. Furthermore, while *t*_0_ and *ϕ* may depend on the initial position *z*_0_ in general, we shall prove later in Theorem 1 that it is indeed independent of *z*_0_.

According to the definition, a QSPS *q*(*t*, *z*) maintains its shape quasi-periodically in *z* variable (with only possible phase change and time shift). In the following, we will claim that the QSPSs defined in Definition 1 have a minimum period, unless for the special case when the signal is a soliton.

To see this, let ${\mathscr{P}}(q)\triangleq \{p:\,p > 0$ such that *q*(*t*, *z*) satisfies (3)}. We call ${\rm{\inf }}\,{\mathscr{P}}(q)$ its minimum period. In this paper, we only consider a QSPS which has a positive minimum period^1^.^1^

#### **Lemma 1**.

*If* ${\rm{\inf }}\,{\mathscr{P}}(q) > 0$, *then the minimum of* ${\mathscr{P}}(q)$ *exists* (*i*.*e*., ${\rm{\inf }}\,{\mathscr{P}}(q)\in {\mathscr{P}}(q)$).

#### *Proof*.

To see this, first notice that if the minimum of ${\mathscr{P}}(q)$ does not exist, then we can construct a strictly decreasing sequence of positive real numbers ${p}_{n}\in \{{\mathscr{P}}(q):\,n=1,2,\ldots \}$ such that ${p}_{n}\to {\rm{\inf }}\,{\mathscr{P}}(q)$, as $n\to \infty$. Next, we will show that ${p}_{m}-{p}_{m+1}\in {\mathscr{P}}(q)$ for all $m\in {{\mathbb{N}}}^{+}$.

First, since *q*(*t*, *z*) is a QSPS with periods *p*_*m*_ and *p*_*m*+1_, for any ${z}_{0}\in {\mathbb{R}}$, there exist *ϕ*_*m*_, *ϕ*_*m*+1_, *t*_*m*_ and *t*_*m*+1_ such that

4 $$q(t,{z}_{0})=q(t-{t}_{m+1},{z}_{0}-{p}_{m+1}){e}^{j{\varphi }_{m+1}}$$

and

5 $$q(t,{z}_{0}+{p}_{m}-{p}_{m+1})=q(t-{t}_{m},{z}_{0}-{p}_{m+1}){e}^{j{\varphi }_{m}}.$$

Now, notice that by (4), we have

6 $$q(t+{t}_{m+1}-{t}_{m},{z}_{0}){e}^{-j{\varphi }_{m+1}}=q(t-{t}_{m},{z}_{0}-{p}_{m+1}).$$

Substitute (6) into (5), we have

7 $$q(t,{z}_{0}+{p}_{m}-{p}_{m+1})=q(t+{t}_{m+1}-{t}_{m},{z}_{0}){e}^{j({\varphi }_{m}-{\varphi }_{m+1})}.$$

Thus, *q*(*t*, *z*) also has a period of *p*_*m*_ − *p*_*m*+1_.

As ${\mathrm{lim}}_{m\to \infty }\,({p}_{m}-{p}_{m+1})=0$, this implies ${\rm{\inf }}\,{\mathscr{P}}(q)=0$ and a contradiction occurs.$\square$

Hence, as long as the infimum of ${\mathscr{P}}(q)$ is positive, it must have a minimum. Throughout this paper, *q*’s minimum period refers to ${\rm{\inf }}\,{\mathscr{P}}(q)$, while its period is any element in ${\mathscr{P}}(q)$.

#### **Lemma 2**.

*Let q*(*t*, *z*) *be a QSPS with minimum period p*. *Then any period of q*(*t*, *z*) *must be an integer multiple of p*.

#### *Proof*.

Let *p*_1_ be a period of *q*(*t*, *z*). Denote *p*_1_ = *np* + *l*, where $n\in {\mathbb{Z}}$, and 0 ≤ *l* < *p*. Suppose to the contrary that *l* ≠ 0. By Definition 1, it is clear that $np\in {\mathscr{P}}(q)$. So $l\in {\mathscr{P}}(q)$ according to (7), which in turn indicates that *l* is a period that is smaller than the minimum period. A contradiction thus arises and the lemma is proved.$\square$

#### Properties of Quasi-Spatially Periodic Signals

We now study the properties of QSPSs. In particular, we study an equivalent definition of QSPSs. We also show the composition of their spectra, and how do the eigenvalues relate to the minimum period. We first review some properties of the NFT^6^.

##### **Proposition 1**

 (NFT properties^6^). *Let ϕ*, *t*_0_ *be real constants* (*ϕ is independent of t*, *but might depend on z*). *Denote u*(*t*, *z*) = *e*^*jϕ*^*q*(*t*, *z*) *and v*(*t*, *z*) = *q*(*t* − *t*_0_, *z*). *Then*

1. (*Spatial evolution*): ${Q}^{(c)}(\lambda ,z)={e}^{-4j{\lambda }^{2}z}{Q}^{(c)}(\lambda ,0)$, ${Q}^{(d)}({\zeta }_{k},z)={e}^{-4j{\zeta }_{k}^{2}z}{Q}^{(d)}({\zeta }_{k},0)$. *The eigenvalues ζ*_*k*_ *keep invariant*, *k* = 1, 2, …, *N*;
2. (*Constant phase change*): *The discrete eigenvalues of u*(*t*, *z*) *and q*(*t*, *z*) *are the same*, *and U*^(*c*)^(*λ*, *z*) = *e*^−*jϕ*^*Q*^(*c*)^(*λ*, *z*), *U*^(*d*)^(*ζ*_*k*_, *z*) = *e*^−*jϕ*^*Q*^(*d*)^(*ζ*_*k*_, *z*);
3. (*Time shift*): *The discrete eigenvalues of v*(*t*, *z*) *and q*(*t*, *z*) *are the same*, *and* ${V}^{(c)}(\lambda ,z)={e}^{-2j\lambda {t}_{0}}{Q}^{(c)}(\lambda ,z)$, ${V}^{(d)}({\zeta }_{k},z)={e}^{-2j{\zeta }_{k}{t}_{0}}{Q}^{(d)}({\zeta }_{k},z)$;
4. (*Parseval*’*s identity*): ${\int }_{-\infty }^{\infty }\,|q(t,0){|}^{2}dt=\widehat{E}+\tilde{E}$, *where* $\widehat{E}=\frac{1}{\pi }\,{\int }_{-\infty }^{\infty }\,\mathrm{log}(1+|{Q}^{(c)}(\lambda ,0){|}^{2})d\lambda$, $\tilde{E}=4\,{\sum }_{k=1}^{N}\,{\rm{Im}}({\zeta }_{k})$.

##### **Theorem 1**.

* Suppose q*(*t*, *z*) *is a QSPS with a period p given in* (3). *Then the time shift t*_0_ *and phase change ϕ with respect to a period p are independent of the initial position z*_0_.

##### *Proof*.

 If *q*(*t*, *z*) is a zero signal, then its time shift and phase change are both arbitrary, and hence are independent of the initial position *z*_0_ considered.

If *q*(*t*, *z*) is not the trivial zero signal, then using the first argument of Proposition 1, for any ${z}_{0}\in {\mathbb{R}}$, we have

8 $${Q}^{(d)}({\zeta }_{i},{z}_{0}+p)={e}^{-4j{\zeta }_{i}^{2}p}{Q}^{(d)}({\zeta }_{i},{z}_{0}).$$

Following (3), and using the second and third arguments of Proposition 1, for any ${z}_{0}\in {\mathbb{R}}$, we have

9 $${Q}^{(d)}({\zeta }_{i},{z}_{0}+p)={e}^{-j\varphi }{e}^{-2j{\zeta }_{i}{t}_{0}({z}_{0})}{Q}^{(d)}({\zeta }_{i},{z}_{0}).$$

Then by (8) and (9), for any ${z}_{0}\in {\mathbb{R}}$, we have ${e}^{-4j{\zeta }_{i}^{2}p}={e}^{-j\varphi -2j{\zeta }_{i}{t}_{0}({z}_{0})},\,i=1,2,\ldots ,N$. So for every ${z}_{0}\in {\mathbb{R}}$ and ${\zeta }_{i}\in {{\mathbb{C}}}^{+}$, there exists an $l(i,{z}_{0})\in {\mathbb{Z}}$ such that $-4j{\zeta }_{i}^{2}p=-\,j\varphi -2j{\zeta }_{i}{t}_{0}({z}_{0})+j2\pi l(i,{z}_{0})$. Let ${\zeta }_{i}\triangleq {\alpha }_{i}+j{\beta }_{i}$, where *β*_*i*_ > 0, $i=1,2,\ldots ,N$. We have

$$4({\alpha }_{i}^{2}-{\beta }_{i}^{2})p+8j{\alpha }_{i}{\beta }_{i}p=\varphi +2{\alpha }_{i}{t}_{0}({z}_{0})+2j{\beta }_{i}{t}_{0}({z}_{0})-2\pi l(i,{z}_{0}).$$

As a result, we have

10 $$4({\alpha }_{i}^{2}-{\beta }_{i}^{2})p=\varphi +2{\alpha }_{i}{t}_{0}({z}_{0})-2\pi l(i,{z}_{0}).$$

and 8*jα*_*i*_*β*_*i*_*p* = 2*jβ*_*i*_*t*_0_(*z*_0_), which gives us

11 $${\alpha }_{i}=\frac{{t}_{0}({z}_{0})}{4p},\,i=1,2,\ldots ,N.$$

Since the eigenvalues *ζ*_*i*_ are invariant during spatial evolution, their real parts *α*_*i*_ and imaginary parts *β*_*i*_ are the same for all *z*_0_. As a result, for any given period *p*, *t*_0_ is also independent of *z*_0_. Similarly, according to equations (10) and (11), we have

12 $${\beta }_{i}^{2}=-\,\frac{\varphi }{4p}-\frac{{t}_{0}^{2}}{16{p}^{2}}+\frac{\pi }{2p}{l}_{i},$$

where ${l}_{i}\triangleq l(i,{z}_{0})\in {\mathbb{Z}}$. So the term −$\frac{\varphi }{4p}+\frac{\pi }{2p}{l}_{i}$ must be independent of *z*_0_. So *ϕ*/4*p* is invariant of *z*_0_ in the sense of mod *π*/2*p*, and hence *ϕ* (mod 2*π*) is *z*_0_-invariant.$\square$

According to Theorem 1, we prove that the time shift *t*_0_ and phase change *ϕ* do not depend on the initial position ${z}_{0}\in {\mathbb{R}}$. This result has an important role of easing the identification of the total time shift and phase change at the receiver. For example, as to be shown in (14) proved in the following corollary, rather than an unknown function, we know the timing centre is shifted *k* times of the time shift for one period of distance *p*, if the total propagation distance is *k* multiples of *p*. Using Theorem 1 we have the following equivalent description of a QSPS.

##### **Corollary 1**.

* A signal q*(*t*, *z*) *propagated in an optical fibre is a QSPS with a period p* > 0 *if and only if there exist real numbers t*_0_ *and ϕ*, *such that*

13 $$q(t,{z}_{0}+p)=q(t-{t}_{0},{z}_{0}){e}^{j\varphi },$$

*and furthermore*,

14 $$q(t,{z}_{0}+kp)=q(t-k{t}_{0},{z}_{0}){e}^{jk\varphi }$$

*for any integer k*, *and real numbers t and z*_0_.

##### *Proof*.

 Equation (13) is a direct consequence of Theorem 1 using Definition 1. Equation (14) can be proved by mathematical induction of (3) because neither *t*_0_ nor *ϕ* depend on the initial position.$\square$

##### **Theorem 2**.

* Let q*(*t*, *z*) *be a signal propagated in an optical fibre*. *Suppose it has discrete spectrum*, *and is denoted by* $\{{\zeta }_{1},{\zeta }_{2},\ldots ,{\zeta }_{N}\}$. *Then the following three statements are equivalent*:

1. *q*(*t*, *z*) *is a QSPS with period p* (*not necessary the minimum period*) *given in* (3);
2. *The continuous spectrum of q*(*t*, *z*) *is null*, *and for* $i=1,2,\ldots ,N$, *its discrete eigenvalues satisfy*
  15 $${\rm{Re}}({\zeta }_{i})=\frac{{t}_{0}}{4p},$$
  *and*
  16 $$|{\zeta }_{i}{|}^{2}\equiv \frac{2\pi -\varphi }{4p}\,({\rm{mod}}\,\frac{\pi }{2p}),\,\varphi \in [0,2\pi ).$$
3. *Its continuous spectrum is null*. *For* $i=1,2,\ldots ,N$, *the real parts of the eigenvalues are identical as given in* (15), *and for each pair of* $m\ne n$,

17 $${a}_{mn}\triangleq \frac{2p\,|{\rm{Im}}{({\zeta }_{m})}^{2}-{\rm{Im}}{({\zeta }_{n})}^{2}|}{\pi }$$

*are positive integers*.

*Furthermore*, *suppose that q*(*t*, *z*) *satisfies* *1*) *and hence also* *3*). *Then p is the minimum period if and only if*

18 $${\rm{\gcd }}({a}_{mn}:\,m\ne n,\,m,n=1,2,\ldots ,N)=1.$$

##### *Proof*.

 We first prove that 1) implies 2). To show the continuous spectrum is null, we only need to prove that ${Q}^{(c)}(\lambda ,0)\equiv 0$, for all $\lambda \in {\mathbb{R}}$. Similar to the method of obtaining (8) and (9) (following (3) and Proposition 1), we have

19 $$\begin{array}{rcl}{Q}^{(c)}(\lambda ,p) & = & {e}^{-4j{\lambda }^{2}p}{Q}^{(c)}(\lambda ,0)\end{array}$$

20 $$\,\begin{array}{rcl} & = & {e}^{-j\varphi }{e}^{-2j\lambda {t}_{0}}{Q}^{(c)}(\lambda ,0).\end{array}$$

Suppose to the contrary that *Q*^(*c*)^(*λ*, 0) is not zero for all $\lambda \in {\mathbb{R}}$, i.e., there exists a real number ${\lambda }_{0}\in {\mathbb{R}}$ such that *Q*^(*c*)^(*λ*_0_, 0) ≠ 0. Since *Q*^(*c*)^(*λ*, 0) is a continuous function in $\lambda \in {\mathbb{R}}$, there exists an interval (*x*_1_, *x*_2_) containing *λ*_0_ such that *Q*^(*c*)^(*λ*, 0) ≠ 0, *λ* ∈ (*x*_1_, *x*_2_). Then by (19) and (20), ${e}^{-4j{\lambda }^{2}p}={e}^{-j\varphi -2j\lambda {t}_{0}}$, *λ* ∈ (*x*_1_, *x*_2_). On the other hand, for every *λ* ∈ (*x*_1_, *x*_2_), there exists an $m(\lambda )\in {\mathbb{Z}}$ such that −4*jλ*^2^*p* = −*jϕ* − 2*jλt*_0_ + *j*2*πm*(*λ*). So, we have

$${(\lambda -\frac{{t}_{0}}{4p})}^{2}=\frac{\varphi }{4p}-\frac{\pi }{2p}m(\lambda )+\frac{{t}_{0}^{2}}{16{p}^{2}},$$

or equivalently,

21 $$\frac{2p}{\pi }({\lambda }^{2}-\frac{{t}_{0}}{2p}\lambda -\frac{\varphi }{4p})=-\,m(\lambda ).$$

However, one can easily find a *λ* ∈ (*x*_1_, *x*_2_) such that the left hand side of (21) is not an integer (could even be an irrational number). In that case, there does not exist any integer *m*(*λ*) satisfying (21), leading to a contradiction. Consider its discrete spectrum, for $i=1,2,\ldots ,N$, equation (15) is proved in Theorem 1 (as in (11)). According to Theorem 1, using (12) and (15), we have (16).

Next, we prove that 2) implies 3). According to (16), we have (12). So we have

$${\rm{Im}}{({\zeta }_{m})}^{2}-{\rm{Im}}{({\zeta }_{n})}^{2}={\beta }_{m}^{2}-{\beta }_{n}^{2}=\frac{\pi }{2p}({l}_{m}-{l}_{n}).$$

Using (17), we have

$${a}_{mn}=\frac{2p\,|{\rm{Im}}{({\zeta }_{m})}^{2}-{\rm{Im}}{({\zeta }_{n})}^{2}|}{\pi }=|{l}_{m}-{l}_{n}|\ge 0.$$

Since *l*_*m*_ ≠ *l*_*n*_ if ${\rm{Im}}({\zeta }_{m})\ne {\rm{Im}}({\zeta }_{n})$ according to (12), 3) is proved.

Now, we prove that 3) implies 1). If the spectrum of a signal *q*(*t*, *z*) satisfies (15) and (17), we have

22 $${e}^{4j{\rm{Im}}{({\zeta }_{m})}^{2}p}={e}^{4j{\rm{Im}}{({\zeta }_{1})}^{2}p},\,m=1,2,\ldots ,N.$$

Denote $x(t,z)\triangleq {e}^{-4j|{\zeta }_{1}{|}^{2}p}q(t-{t}_{0},z),\,t\in {\mathbb{R}},\,z\in {\mathbb{R}}$. Then according to Proposition 1, *x*(*t*, *z*) and *q*(*t*, *z*) have the same discrete eigenvalues because the former is just a constant phase change and time shift of the latter. In addition, for $i=1,2,\ldots ,N$,

23 $$\begin{array}{rcl}{X}^{(d)}({\zeta }_{i},z) & = & {e}^{4j|{\zeta }_{1}{|}^{2}p}{e}^{-2j{\zeta }_{i}{t}_{0}}{Q}^{(d)}({\zeta }_{i},z)\\  & = & {e}^{4j[{\rm{Im}}{({\zeta }_{1})}^{2}-{\rm{Re}}{({\zeta }_{i})}^{2}-2j{\rm{Re}}({\zeta }_{i}){\rm{Im}}({\zeta }_{i})]p}{Q}^{(d)}({\zeta }_{i},z)\end{array}$$

24 $$\begin{array}{rcl} & = & {e}^{4j[{\rm{Im}}{({\zeta }_{i})}^{2}-{\rm{Re}}{({\zeta }_{i})}^{2}-2j{\rm{Re}}({\zeta }_{i}){\rm{Im}}({\zeta }_{i})]p}{Q}^{(d)}({\zeta }_{i},z)\\  & = & {Q}^{(d)}({\zeta }_{i},z+p)\end{array}$$

where (23) is true according to (15), and (24) is obtained by (22). Since its continuous spectrum is null, we have

$$q(t,z+p)=x(t,z)={e}^{-4j|{\zeta }_{1}{|}^{2}p}q(t-{t}_{0},z)$$

for any $t\in {\mathbb{R}}$, and $z\in {\mathbb{R}}$. So *q*(*t*, *z*) is a QSPS with period *p* according to Definition 1.

Now, it remains to prove that *p* is minimum if and only if (18) holds. Hence, *p* is the minimum period if and only if *p* is the smallest positive real number such that

$${a}_{mn}\triangleq \frac{2p|{\rm{Im}}{({\zeta }_{m})}^{2}-{\rm{Im}}{({\zeta }_{n})}^{2}|}{\pi }$$

are integers for all distinct *m*, *n*. Let $x\triangleq {\rm{\gcd }}({a}_{mn}:\,m\ne n,\,m,n=1,2,\ldots ,N)$. So for any distinct *m*, *n*,

$${a}_{mn}^{^{\prime} }\triangleq \frac{{a}_{mn}}{x}=\frac{2(p/x)\,|{\rm{Im}}{({\zeta }_{m})}^{2}-{\rm{Im}}{({\zeta }_{n})}^{2}|}{\pi }$$

is still an integer. Consequently, if *p* is the minimal period, then *x* = 1. On the other hand, if *p* is not the minimum period, then by Lemma 2, *p* = *cp*′ where *p*′ is the minimum period and *c* is an integer greater than 1. It can be verified directly that *x* = *c* > 1. Hence, we prove the theorem.$\square$

Theorem 2 analyses the spectral components of a QSPS, which is rather simple. It lets us know that a QSPS is equivalent to a spectral domain signal with null continuous spectrum, and its discrete eigenvalues are located in a special manner. Because of the null continuous spectrum, Darboux transform^7^ is enough to convert the spectral domain signal to its time domain representation, avoiding the computational more expensive Riemann-Hilbert system^6^. This theorem leads us explicitly how to design a QSPS, and check whether a spectral domain signal is a QSPS. To be more specific, Theorem 2 implies that all QSPSs are *N*-solitons. However, its converse is not true.

##### **Remark 1**.

* According to Theorem 2*, *the eigenvalues of a QSPS should satisfy*

25 $$\frac{|{\rm{Im}}{({\zeta }_{m})}^{2}-{\rm{Im}}{({\zeta }_{n})}^{2}|}{|{\rm{Im}}{({\zeta }_{l})}^{2}-{\rm{Im}}{({\zeta }_{m})}^{2}|}=\frac{{a}_{mn}}{{a}_{lm}}\in {\mathbb{Q}}$$

*for any l* ≠ *m* ≠ *n*, $l,m,n\in \{1,2,\ldots ,N\}$ *when N* ≥ 3. Equation (25) *gives us an insight that the eigenvalues cannot be chosen arbitrarily* (*even if all their real parts are the same*) *if quasi*-*spatially periodic property is required when there are more than two discrete eigenvalues*.

##### **Example 1**

 (A non-quasi-spatially-periodic *N*-soliton). *Let N* = 3, *and* ${\zeta }_{1}=\sqrt[3]{2}j$, *ζ*_2_ = 2*j*, *ζ*_3_ = 3*j*. *We have*

$$\frac{|{\rm{Im}}{({\zeta }_{2})}^{2}-{\rm{Im}}{({\zeta }_{3})}^{2}|}{|{\rm{Im}}{({\zeta }_{1})}^{2}-{\rm{Im}}{({\zeta }_{2})}^{2}|}=\frac{5}{4-\sqrt[3]{4}}\notin {\mathbb{Q}}.$$

*As a result*, $\{\sqrt[3]{2}j,2j,3j\}$ *is not the spectrum of any QSPS*.

#### A Spectral Domain Interpretation of Quasi-Periodicity

So far, we define the quasi-periodicity of a QSPS in the time domain (Definition 1 and Corollary 1) as that the signal maintains its shape quasi-periodically with a phase change and a time shift. In the following, we study the quasi-periodicity in the spectral domain, showing that phase changes of all the spectral amplitudes agree with each other periodically.

##### **Definition 2**.

* The* “*spectral period*” *induced by two distinct eigenvalues of a QSPS is one of the distances at which the phase changes of these two corresponding discrete spectral amplitudes in their spectral domain spatial evolution are identical*. *More precisely*, *the spectral periods induced by two distinct eigenvalues* (*ζ*_*m*_ *and ζ*_*n*_, *m* ≠ *n*) *are elements of the set*

26 $$\{z > 0:\,{e}^{-4j{\rm{Re}}({\zeta }_{m}^{2})z}={e}^{-4j{\rm{Re}}({\zeta }_{n}^{2})z}\}.$$

*We denote the minimum spectral period of ζ*_*m*_ *and ζ*_*n*_ *by p*(*ζ*_*m*_,*ζ*_*n*_).

Similar to the discussion after Definition 1, we can show that *p*(*ζ*_*m*_,*ζ*_*n*_) exists for any *m* ≠ *n*.

##### **Corollary 2**.

* Phase changes of any two spectral amplitudes of a QSPS during the spatial evolution along an optical fibre coincide periodically*. *Specifically*, *the minimum spectral period for ζ*_*m*_ *and ζ*_*n*_ (*m* ≠ *n*) *is*

27 $$p({\zeta }_{m},{\zeta }_{n})=\frac{\pi }{2\,|{\rm{Im}}{({\zeta }_{m})}^{2}-{\rm{Im}}{({\zeta }_{n})}^{2}|}=\frac{p}{{a}_{mn}},$$

*where p is the minimum period when* (18) *is satisfied*.

##### *Proof*.

 According to Proposition 1 and (26), for *m* ≠ *n*, the corresponding spectral amplitudes have the same phase change when ${e}^{-4j[{\rm{Re}}{({\zeta }_{m})}^{2}-{\rm{Im}}{({\zeta }_{m})}^{2}]z}={e}^{-4j[{\rm{Re}}{({\zeta }_{n})}^{2}-{\rm{Im}}{({\zeta }_{n})}^{2}]z}$, where $z\in {\mathbb{R}}$, which is equivalent to $4{\rm{Im}}{({\zeta }_{m})}^{2}z\equiv 4{\rm{Im}}{({\zeta }_{n})}^{2}z$ $({\rm{mod}}\,2\pi )$ according to Theorem 2, which is further equivalent to

$$z\equiv 0\,({\rm{mod}}\,\frac{\pi }{2\,|{\rm{Im}}{({\zeta }_{m})}^{2}-{\rm{Im}}{({\zeta }_{n})}^{2}|}).$$

According to Theorem 2, we have (27), where *p* is the minimum spectral period when (18) is satisfied.$\square$

As a simple observation from Corollary 2, if a QSPS only has two eigenvalues *ζ*_1_ and *ζ*_2_ with minimum period *p*, we have *p* = *p*(*ζ*_1_,*ζ*_2_), because *a*_12_ = 1 observed from (18). This also shows that the period of a QSPS (in the spectral domain) is the minimal integer multiple of the minimum spectral period for any pair of eigenvalues.

### Trade-off between Energy, Minimum Period and Degree-of-Freedom

So far, we have studied the properties of QSPSs. In particular, we obtain the relationship between the eigenvalues and the minimum period of a QSPS. In the following, we also take energy into consideration, and consider the signal design for QSPSs. Specifically, we derive a trade-off between various parameters including minimum period, signal energy and degree-of-freedom. Note that we refer degree-of-freedom of a QSPS as the number of its discrete eigenvalues, as it is often the number of users allowed for interference free multiuser communications under the NFDM scheme. In this subsection, we call a QSPS with *N* eigenvalues an *N*-QSPS.

#### **Theorem 3**.

*Let q*(*t*, *z*) *be an N*-*QSPS with minimum period p and energy E*. *If E is large enough* (*E satisfies* (29)), *there exists a positive real number β*_1_ (*which is the smallest among all the imaginary parts of the eigenvalues*) *and integers* ${a}_{s},s=1,2,\ldots ,N-1$ *such that*

28 $$\frac{E}{4}={\beta }_{1}+\sum _{k=2}^{N}\,\sqrt{{\beta }_{1}^{2}+\frac{\pi }{2p}\,\sum _{s=1}^{k-1}\,{a}_{s}}.$$

#### *Proof*.

Let the discrete spectrum of *q*(*t*, *z*) be $\{{\zeta }_{1},{\zeta }_{2},\ldots ,{\zeta }_{N}\}$. By Theorem 2, all the discrete eigenvalues have the same real parts. Therefore, we denote that *ζ*_*k*_ = *α* + *jβ*_*k*_ with *β*_*k*_ > 0 for $k=1,\ldots ,N$. In fact, we assume without loss of generality that ${\beta }_{1} < {\beta }_{2} < \cdots  < {\beta }_{N}$. Let ${a}_{s}=\frac{2p({\beta }_{s+1}^{2}-{\beta }_{s}^{2})}{\pi }$, $s=1,2,\ldots ,N-1$. By Theorem 2, *a*_*s*_ is an integer. In addition, ${\beta }_{k}^{2}-{\beta }_{1}^{2}=({\sum }_{s=1}^{k-1}\,{a}_{s})\frac{\pi }{2p}$ for $k=2,3,\ldots ,N$. Hence,

$${\beta }_{k}=\sqrt{{\beta }_{1}^{2}+\frac{\pi }{2p}\,\sum _{s=1}^{k-1}\,{a}_{s}},\,k=2,3,\ldots ,N.$$

Finally, (28) follows from Proposition 1.$\square$

When we design an optical system, *N* is the degree-of-freedom and can be regarded as the number of subchannels. Roughly speaking, the larger the *N* is, the more information can be transmitted across to the receiver. On the other hand, since *p* is the minimum period, the transmitter and the receiver must be separated by a distance of an integer multiple of *p*. Therefore, it is desirable to minimise *p* for the sake of flexibility. The natural question thus is: Can we design a system which minimises *p* and maximises *N* simultaneously? Unfortunately, the answer is no according to the next corollary.

#### **Corollary 3**

(Trade-off). *Let q*(*t*, *z*) *be an N*-*QSPS with minimum period p*, *energy E and degree*-*of*-*freedom N*. *Then*

29 $$E > 4\sqrt{\frac{\pi }{2p}}\,(\sum _{k=1}^{N-1}\,\sqrt{k}).$$

*Consequently*, *we cannot design a communication system which minimises minimum period*, *and maximises the number of subchannels at the same time using QSPSs*.

#### *Proof*.

Let *q*(*t*, *z*) be quasi-spatially periodic with minimum period *p*, energy *E* and *N* eigenvalues. According to Theorem 3 (equation (28)), *E* can be minimised by minimising *β*_1_ and also *a*_*s*_. Clearly, it is required that *β*_1_ > 0 and ${a}_{1},\ldots ,{a}_{N-1}$ satisfying (18). Let *a*_*s*_ = 1 for all $s=1,2,\ldots ,N-1$. It can be checked easily that (29) is true. In fact, for any *ε* > 0, one can easily construct a QSPS such that

$$E=4\sqrt{\frac{\pi }{2p}}\,(\sum _{k=1}^{N-1}\,\sqrt{k})+\varepsilon .$$

$$\square $$

Theorem 3 tells us that constructing a QSPS with minimum period *p* and energy *E* is equivalent to solving the equation (28). In fact, we can always find an *N*-QSPS with minimum period *p* and energy *E* as long as (29) holds.

When *N* = 2, the choice of the imaginary parts of the eigenvalues is in fact unique:

$${\beta }_{1}=j\,(\frac{1}{8}E-\frac{\pi }{pE}),\,{\beta }_{2}=j\,(\frac{1}{8}E+\frac{\pi }{pE})$$

if and only if (29) holds for *N* = 2. However, the uniqueness may not hold in general when *N* > 2.

Theorem 3 provides us a starting point of designing QSPSs. Actually, besides minimum period, energy and degree-of-freedom, there are many other factors affecting signal design. If noise is taken into consideration, we often do not expect the SNR to be low. So the *β*_1_ in (28) should not be chosen very small. In addition, since the signal detection at the receiver relies on the quasi-periodicity of the signal, the position of the detector is subtle. As a result, we expect signals whose shape do not change a lot within a small difference of position. Moreover, we prefer signals to have short duration to increase the efficiency of the communication system, which is also relevant to the choice of signal shape. Theorem 3 provides us an insight that we still have much choices to find optimal pulse shapes for information transmission to obtain a better performance even if the minimum period and energy are fixed when the signals have more than two eigenvalues.

### Pulse Truncation

So far, we have studied properties and design of QSPSs. In practice, signals are always time-limited (i.e., with finite duration). Therefore, when time-unlimited QSPSs are transmitted in practice, they are often “truncated”. However, truncation causes the discrete eigenvalues to be distorted leading to that nontrivial continuous spectrum would appear. So it is interesting to study how the “quasi-periodicity” of the effect QSPSs will be affected by truncation.

**Figure 1:**
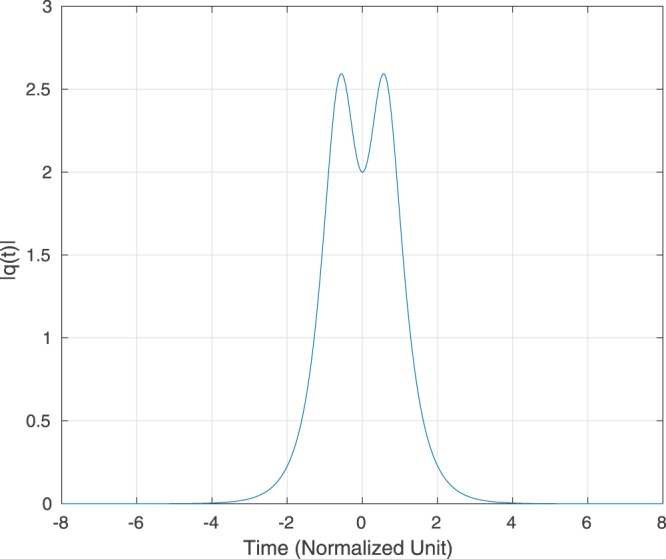
Magnitude of the QSPS *q*(*t*, 0) with Eigenvalues (*j*, 2*j*).

To achieve the goal, we ran a simulation for the noiseless propagation of a “truncated QSPS”. In this example, we consider a QSPS which has eigenvalues {*j*, 2*j*} and corresponding spectral amplitudes both equal to 1. Also, its period is $p=\frac{\pi }{6}\approx 0.52360$, according to Theorem 2. We first implement the INFT using the Darboux transformation^8^ to obtain the time domain representation of the signal with the time support [−8, 8] and denote it as *q*(*t*, 0), whose magnitude is plotted in Figure 1. We then truncate *q*(*t*, 0) such that it is strictly time limited in [−2.5, 2.5]. Denote the truncated pulse by *q*_trun_(*t*, 0). The truncated pulse *q*_trun_(*t*, 0) is then sent to a simulated fibre of normalized distance *L* = 2. Note also that *L* > 3*p*. Then we compute the NFT of *q*_trun_(*t*, 0), and its eigenvalues are (0.998*j*, 1.999966*j*). The result agrees with^15^ that the smaller eigenvalue is distorted more severely than the bigger one. According to Theorem 2, the period of *q*_trun_(*t*, 0) is *p*_trun_ = 0.52297.

**Figure 2:**
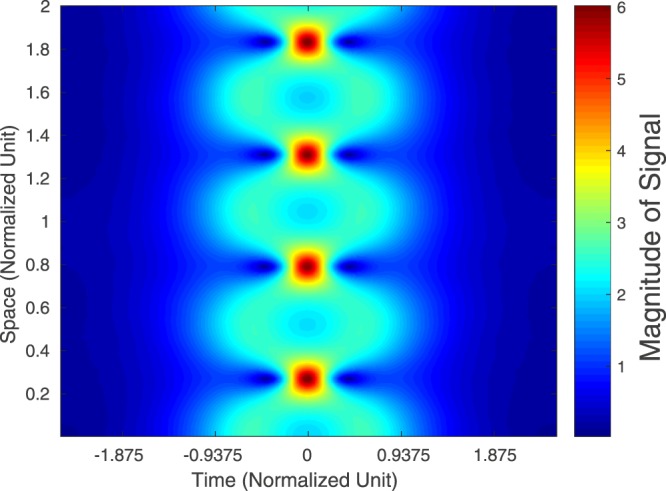
Heatmap of the Magnitude of the Truncated QSPS with Eigenvalues (*j*, 2*j*) as Propagated.

**Figure 3:**
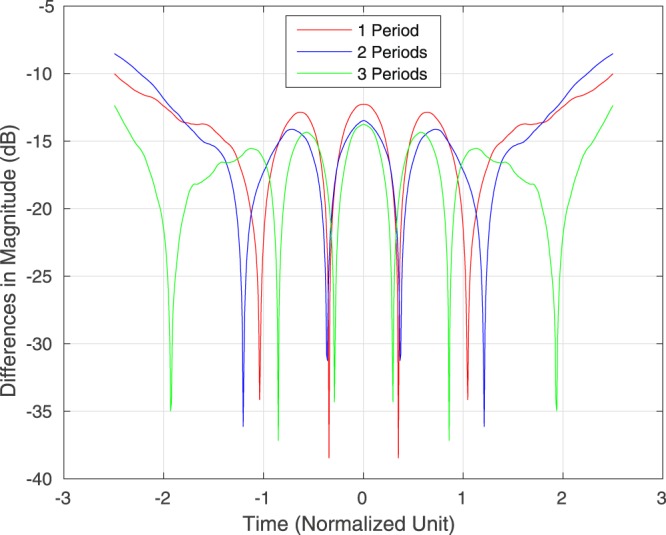
Difference Between the Pulse in Each Period of Distance and the Transmitted Truncated QSPS with Eigenvalues (*j*, 2*j*).

As before, we let *q*_trun_(*t*, *z*) and *q*(*t*, *z*) be the signals obtained by respectively propagating *q*_trun_(*t*, 0) and *q*(*t*, 0) for a distance of *z* along the fibre. Then Figure 2 is the heatmap of |*q*_trun_(*t*, *z*)|, showing that the “truncated QSPS” is roughly quasi-periodic. Figure 3 shows the absolute difference |*q*_trun_(*t*, *z*) − *q*(*t*, *z*)| when *z* = *kp*_trun_ for *k* = 1, 2, 3. In most of the times, the difference is within −10 dB. Our simulations also show that further truncation of *q*(*t*, 0) will increase the error.

## Discussion

In this paper, we study a class of signals, which have the quasi-spatially periodic property. We show that all QSPSs have a null continuous spectrum, and the property of quasi-spatially periodic only depends on their eigenvalues, but not spectral amplitudes. The results are of theoretical importance because they help understand the structure of their spectra for further use as input signals in optical fibre communication systems.

In addition, the property of quasi-spatially periodic motivates us a novel idea of decoding that could possibly replace the costly NFT. Specifically, the minimum period of a QSPS could be designed beforehand such that the same shape is received at the output with only a phase change and possibly a time shift. In high SNR regime, it is expected that time domain signal processing technique replace the role of the NFT for decoding with the knowledge of the shapes of signals. Having the property of quasi-spatially periodic, it is beneficial to achieve this goal for optical fibre communications.

## Methods

Mathematical derivation and proofs, as well as numerical simulation are the methods used in this paper. Proofs are given for all results presented in this paper.

## Footnotes

1 ^1^If ${\rm{\inf }}\,{\mathscr{P}}(q)=0$, then it can be easily checked that the QSPSs (3) degenerate to fundamental solitons.
